# SNP-SNP Interaction between TLR4 and MyD88 in Susceptibility to Coronary Artery Disease in the Chinese Han Population

**DOI:** 10.3390/ijerph13030278

**Published:** 2016-03-04

**Authors:** Dandan Sun, Liping Sun, Qian Xu, Yuehua Gong, Honghu Wang, Jun Yang, Yuan Yuan

**Affiliations:** 1 Department of Tumor Etiology and Screening, Cancer Institute and General Surgery, The First Affiliated Hospital of China Medical University, Shenyang 110001, China; dan_101912@163.com (D.S.); lipingsun@hotmail.com (L.S.); xuqian820101@163.com (Q.X.); gongyueh75@163.com (Y.G.); 2 Key Laboratory of Cancer Etiology and Prevention, Liaoning Provincial Education Department, China Medical University, Shenyang 110001, China; 3 Department of Cardiovascular Ultrasound, The First Affiliated Hospital of China Medical University, Shenyang 110001, China; wanghonghuzgykd@126.com (H.W.); junyang63@sina.com (J.Y.)

**Keywords:** toll-like receptor 4, myeloid differentiation factor 88, polymorphism, Interaction, coronary artery disease

## Abstract

The toll-like receptor 4 (TLR4)-myeloid differentiation factor 88 (MyD88)-dependent signaling pathway plays a role in the initiation and progression of coronary artery disease (CAD). We investigated SNP–SNP interactions between the *TLR4* and *MyD88* genes in CAD susceptibility and assessed whether the effects of such interactions were modified by confounding risk factors (hyperglycemia, hyperlipidemia and *Helicobacter pylori* (*H. pylori*) infection). Participants with CAD (*n* = 424) and controls (*n* = 424) without CAD were enrolled. Polymerase chain restriction-restriction fragment length polymorphism was performed on genomic DNA to detect polymorphisms in *TLR4* (rs10116253, rs10983755, and rs11536889) and *MyD88* (rs7744). *H. pylori* infections were evaluated by enzyme-linked immunosorbent assays, and the cardiovascular risk factors for each subject were evaluated clinically. The significant interaction between *TLR4* rs11536889 and *MyD88* rs7744 was associated with an increased CAD risk (*p* value for interaction = 0.024). In conditions of hyperglycemia, the interaction effect was strengthened between *TLR4* rs11536889 and *MyD88* rs7744 (*p* value for interaction = 0.004). In hyperlipidemic participants, the interaction strength was also enhanced for *TLR4* rs11536889 and *MyD88* rs7744 (*p* value for interaction = 0.006). Thus, the novel interaction between *TLR4* rs11536889 and *MyD88* rs7744 was related with an increased risk of CAD, that could be strengthened by the presence of hyperglycemia or hyperlipidemia.

## 1. Introduction

Coronary artery disease (CAD) is the most common cause of morbidity and mortality in China [1]. It is a complex disease determined by genetic predisposition and environmental factor accumulation, which play major roles in a number of associated vessel wall abnormalities [2]. A person’s genetic make-up as well as other well-known major risk factors are important for the initiation and progression of CAD. Indeed, a substantial body of literature has investigated the association of CAD with gene polymorphisms [3,4,5].

Toll-like receptor 4 (TLR4) and myeloid differentiation factor 88 (MyD88), which act as the gate of the innate immune system and the trigger of the adaptive immune system, have been extensively studied for their roles in the pathogenesis and progression of CAD [6,7]. Compared to the wild-type mice, the mice deficiency of the *TLR4* gene or *MyD88* gene exhibited significantly smaller infarctions, as well as lower levels of some atherogenic cytokines (e.g., IL-1β, IL-6, and TNFα) [8]. Some studies have found that a coding polymorphism in the *TLR4* gene was associated with CAD or acute myocardial infarction in a Caucasian population, but not in a Chinese population [9]. Regarding the MyD88 gene, a single nucleotide polymorphism (SNP) in its 3′-untranslated region (3′-UTR) has been reported to be associated with Buerger disease but not with Takayasu arteritis in the Japanese population [10]. Up to date, 153 suggestive DNA variants associated with CAD have been discovered by genome-wide association study (GWAS) worldwide. However, each variant usually confers a minimal to modest increase in relative risk, averaging only 18% (corresponding to an odds ratio of 1.18) [11]. Accordingly, the results of genetic polymorphism studies that have sought to identify relationships for *TLR4* and *MyD88* genes with CAD remain controversial and inconclusive. In most studies, the association between the risks of CAD and genetic polymorphisms was often limited to one loci or haplotypes over several neighboring loci in one gene of interest, which seems insufficient as the genetic baseis for CAD is complex and varied [12]. Thus, an increasing number of studies have assessed epistatic gene-gene interaction effects on CAD susceptibility [13,14]. TLR4 is an important membrane receptor, which not only can recognize most of exogenous ligands, like lipopolysaccharide (LPS) of *Helicobacter pylori* (*H. pylori*) [15], but also can bind to some endogenous ligands, such as fetuin-A (FetA) related to hyperglycemia and minimally modified low density lipoprotein (mmLDL) involved in hyperlipidemia [16,17]. Thus, we made further efforts on evaluation of the modified function of the related environmental factors to the SNP-SNP interaction effect of *TLR4* and *MyD88* genes that are not.

Consequently, in this study, we investigated potential SNP-SNP interactions of *TLR4* and *MyD88* genes for their possible roles in susceptibility to CAD. We assessed whether the effects of such interactions were modified by environmental factors, such as hyperglycemia, hyperlipidemia and *H. pylori* infection, in order to determine the architecture of CAD predisposition and thereby improve personalized preventative for individuals at risk of this disease.

## 2. Materials and Methods

### 2.1. Study Population

This was a single center, case-control study. We collected data from 848 consecutive participants who had undergone coronary angiography at the First Affiliated Hospital of China Medical University between 2012 and 2015. This study was approved by the Ethics Committee (Ethic Approval Number: [2011]18 and 2015-68-2). Patients who had at least one vessel with stenosis of no less than 50% diameter were defined as CAD cases (*n* = 424). Those who had no demonstrable lesions on angiography served as controls (*n* = 424). The exclusion criteria were as follows: participants with cardiomyopathy, auto-immunological disease, severe kidney or liver disease, or malignant disease.

All participants had their demographic characteristics (e.g., age, sex) recorded and were examined to determine the presence of cardiovascular risk factors. The confounding risk factors were as follows: (a) smoking: individuals who had smoked at least one cigarette per day for more than one year were classified as smokers; (b) alcohol consumption: individuals who had consumed at least one alcoholic drink a day for a minimum period of six months were defined as consumers of alcohol; (c) hypertension: individuals with systolic blood pressure ≥140 mmHg or diastolic blood pressure ≥90 mmHg, or both, were considered hypertensive; (d) hyperglycemia: individuals with fasting plasma glucose ≥7.0 mmol/L or 2-h plasma glucose ≥11.1 mmol/L, or both, were considered hyperglycemic; (e) hyperlipidemia: participants with plasma cholesterol concentration ≥5.17 mmol/L, or plasma triglyceride concentration ≥1.70 mmol/L or plasma low-density lipoprotein cholesterol concentration ≥2.58 mmol/L, were considered hyperlipidemic. Details of the study group characteristics were summarized in Table 1.

### 2.2. SNP Selection and Genotyping

A two-step approach was performed to identify tag-SNPs in *TLR4* and *MyD88* genes as described previously [18]. First, tag-SNPs were selected in the combinations provided by the HapMap database (Release 27, Phase I + II + III) and Haploview software [19,20]. Next, the functional effects of the selected tag-SNPs were predicted by FuncPred software [21]. Accordingly, two tag-SNPs (rs10116253 and rs10983755) in the promoter region of *TLR4*, one tag-SNP (rs11536889) in the 3′-UTR of *TLR4* and one tag-SNP (rs7744) in the 3′-UTR of *MyD88* were screened.

Genomic DNA of each subject was extracted from a blood clot using standard phenol-chloroform methodology. The polymorphisms were detected using the polymerase chain restriction-restriction fragment length polymorphism (PCR-RFLP) procedure. Table S1 shows the details of the PCR-RFLP conditions of the four tag-SNPs.

### 2.3. H. pylori Serology

The concentration of serum IgG, specific for *H. pylori* was tested using an enzyme-linked immunosorbent assay (*H. pylori* IgG ELISA kit; BIOHIT, Helsinki, Finland). The cut-off value is 34 EIU, which is given by the standard protocol (BIOHIT, Helsinki, Finland). If the titer value was above 34 EIU, the individual was defined as *H. pylori* infection [22].

### 2.4. Statistical Analyses

All statistical analyses were performed using the SPSS 16.0 statistical software package (SPSS, Chicago, IL, USA). Discrete variables, represented as frequencies and percentages, were evaluated by Pearson’s χ^2^ tests. Continuous variables, presented as mean ± SD, were compared using ANOVA tests. SNP–SNP interaction effects were assessed using the likelihood-ratio tests, by comparing the fit of the logistic model that included the main effects of the environment risk factors and genotypes with a fully parameterized model [23]. Odds ratios (OR) with 95% confidence intervals (CI) were calculated as measures of associations adjusted by the confounding risk factors (age, sex, hypertension, hyperglycemia, hyperlipidemia and *H. pylori* infection) unless the risk factor had been used as a stratified factor. A two-side *p* value of <0.05 was considered statistically significant.

## 3. Results

### 3.1. Main Effect Analyses of Individual Polymorphisms in the TLR4 and MyD88

The genotype distributions of the four SNPs studied in the control participants followed Hardy-Weinberg equilibrium (HWE) (*p* > 0.05) (Table S2). In our unpublished data, we found that of the polymorphisms in *TLR4* and *MyD88*, the TLR4 rs10116253 polymorphism was associated with a slightly decreased risk of CAD, whereas there was no overall genetic effect for *TLR4* rs10983755, *TLR4* rs11536889 or *MyD88* rs7744 relating to CAD risk.

### 3.2. Two-Way Interactions between TLR4 and MyD88 Polymorphisms

In the two-way interaction analyses, the most significant interaction was between *TLR4* rs11536889 and *MyD88* rs7744. This interaction was associated with an increased risk of CAD (*p* value for interaction = 0.024, OR (95% CI) = 1.928 (1.089–3.413)). In contrast, in the two-way analyses between *TLR4* rs10116253 or *TLR4* rs10983755 and *MyD88* rs7744, no statistically significant interactions were observed (*p* value for interaction >0.05) (Table 2).

### 3.3. The Effect of Confounding Risk Factors on the Interaction between Polymorphisms in TLR4 and MyD88

In stratified analyses, we tested the effect of environmental risk factors (*H. pylori* infection, hyperglycemia and hyperlipidemia) on the interaction strength (Table 3). Under conditions of hyperglycemia, the OR (95% CI) was 4.905 (1.640–14.673) between *TLR4* rs11536889 and *MyD88* rs7744 (*p* value for interaction = 0.004). In contrast, the OR (95% CI) was 1.336 (0.664–2.686) for the participants with normal plasma glucose levels (*p* value for interaction = 0.417). Moreover, when the participants had hyperlipidemia, the OR (95% CI) was 3.269 (1.398–7.644) between *TLR4* rs11536889 and *MyD88* rs7744 (*p* value for interaction = 0.006). However, no interaction effect was noted in the participants who lacked hyperlipidemia (OR (95% CI) = 1.156 (0.513–2.604), *p* value for interaction = 0.726). Furthermore, *H. pylori* infection did not influence the interaction effect between *TLR4* rs11536889 and *MyD88* rs7744 for CAD risk (*p* value for interaction >0.05). As to the analyses between *TLR4* rs10116253 or *TLR4* rs10983755 and MyD88 rs7744, no modification by any of the environmental risk factors was identified (*p* value for interaction >0.05) (Table 4 and Table 5).

## 4. Discussion

Genetic polymorphisms in humans can be used to predict the risks of particular diseases occurring. However, many previous studies have focused their attention on identifying single gene polymorphisms responsible for disease risk, but often no effects or weak effects have been found in such studies [24,25]. Recently, increasing studies have investigated interactions among combinations of two or more SNPs, and the results have usually revealed a moderate or strong effect on disease risk [23,26]. To the best of our knowledge, this study is the first to assess the interaction effects of *TLR4* and *MyD88* polymorphisms on CAD risk in the Chinese Han population. TLR4, as the gate of inflammatory reaction, not only can recognize pathogen-associated molecular patterns (PAMPs), but also can initiate inflammation in the lipid-laden artery wall via the NF-κB pathway, which have been proved to take part in the initiation and progression of atherosclerosis and its related complications [27,28]. As to MyD88, the cytoplasmic receptor adaptor of TLR4, has been widely studied in atherogenesis. Besides involving in the classical TLR4-MyD88-dependent signaling pathway related to atheroscleorsis, MyD88 has been also played an important role in obesity-associated inflammatory diseases, including insulin resistance and atherosclerosis [29]. Hence, we performed interaction effect analyses on three tag-SNPs in *TLR4* (rs10116253, rs10983755 and rs11536889) and one tag-SNP in *MyD88* (rs7744) to evaluate the risk of CAD in the Chinese Han population. We found that an interaction effect between rs11536889 in *TLR4* and rs7744 in *MyD88* was associated with an increased risk of CAD. Furthermore, the interaction effect was exacerbated by the presence of hyperglycemia or hyperlipidemia.

Evidence is accumulating that TLR4 and MyD88 have a close relationship with many inflammation-related diseases, and many studies in recent years have focused on polymorphisms in *TLR4* and *MyD88* genes with disease risk [30,31]. Some researchers have reported that *TLR4* rs11536889 polymorphism is associated with a variety of autoimmune diseases, such as Grave’s disease and autoimmune pancreatitis [32]. A study by Wang *et al.* revealed a relationship between *TLR4* rs11536889 and sepsis [33]. Furthermore, the results from Sato *et al.* indicated that genetic variation of rs11536889 contributes to translational regulation of TLR4, possibly by binding to microRNAs [34]. Regarding *MyD88* rs7744, Chen *et al.* found that the variant genotypes of rs7744 were associated with Buerger’s disease in a Japanese population [10]. However, we found that when analyzed as a single locus, neither *TLR4* rs11536889 nor *MyD88* rs7744 had an effect on CAD risk. In contrast, the interaction effect of *TLR4* rs11536889 and *MyD88* rs7744 was associated with an increased risk of CAD. We consider the interaction effect of these two SNPs to be epistasis, which has been involved in susceptibility to various inflammation-related diseases, such as malignant tumors, asthma, and Parkinson's disease [35,36,37]. The epistatic effect of two or more genes can account for the missing heritability of many diseases, a phenomenon often underestimated or even ignored. Indeed, the genetic effects of *TLR4* rs11536889 and *MyD88* rs7744 polymorphisms on the risk of CAD would most likely have been missed had they not been tested jointly. Consequently, the epistatic effects of TLR4 rs11536889 and MyD88 rs7744 on the pathogenesis and progression of CAD might depend on the presence of the other SNP. It is assumed that a functional effect on TLR4 and MyD88 in the TLR4-MyD88-dependent signaling pathway might account for the interaction effect we observed. Any genetic mutation within this pathway, especially in key genes like *TLR4* and *MyD88*, could potentially alter the action of other components of the pathway so as to influence inflammatory reactions in the pathogenesis and progression of atherosclerosis. Our study focused only on a few tag-SNPs with potential functions in the promoter and 3′-UTR of *TLR4* and *MyD88* genes, but this approach does not capture all genetic variants in these two genes. Therefore, further analyses covering more tag-SNPs should be undertaken to investigate the *potential* interaction effects of *TLR4* and *MyD88* more fully.

In the current study, heterogeneity in the hyperglycemia or hyperlipidemia status of the study participants had a significant effect on the interaction of *TLR4* rs11536889 and *MyD88* rs7744. Moreover, the interaction strength was enhanced under conditions of hyperglycemia or hyperlipidemia. Evidence suggests that exogenous and endogenous ligands can activate the TLR4-MyD88-dependent signaling pathway [38,39]. Miller *et al.* showed that the mmLDL-induced stimulation of macropinocytosis was TLR4 dependent and resulted in lipid accumulation in macrophages [17]. Pal *et al.* found that FetA played a crucial role in regulating insulin sensitivity via the TLR4-MyD88-dependent signaling pathway in mice. FetA knockdown in mice with hyperglycemia resulted in inactivation of the TLR4-MyD88-dependent signaling pathway, whereas selective administration of FetA induced inflammatory signaling and insulin resistance [16]. In addition, Yu *et al.* [29] showed MyD88-dependent interplay between myeloid and endothelial cells in the initiation and progression of atherosclerosis. MyD88 deficiency in endothelial cells results in a moderate reduction in diet-induced adipose macrophage infiltration, and M1 polarization, selective insulin sensitivity in adipose tissue, and amelioration of spontaneous atherosclerosis [29]. Therefore, we hypothesized that TLR4 and MyD88 were highly likely to be associated with hyperglycemia and hyperlipidemia, consistent with the effect-modification by hyperglycemia and hyperlipidemia that was observed in *TLR4* rs11536889 and *MyD88* rs7744 interaction.

Although the LPS of *H. pylori* has been shown to be one of the most powerful exogenous TLR4 ligands, there is no evidence of systemic invasion of Η. pylori beyond the intestinal mucosa. Researchers have looked for *H. pylori* DNA in atheromatous tissue specimens using PCR. Kaklikkaya *et al.* did not detect *H. pylori* DNA in 21 patients with aortoiliac occlusive disease [40]. In addition, Dore *et al.*, found that only one of 32 atherosclerotic plaques obtained at endarterectomy was positive for *H. pylori* DNA; however, the possibility of contamination could not be excluded in this study [41]. Hishiki *et al.* have speculated that a relationship between *H. pylori*, decreased body mass index and decreased plasma total cholesterol caused by dyspepsia exists, and that eradication of *H. pylori* might exaggerate the metabolic syndrome [42]. In the present study, no interaction effect between *TLR4* rs11536889 and *MyD88* rs7744 polymorphisms in the subgroup analyses for *H. pylori* infection was identified. Taken together, the evidence above indicates that *H. pylori* is unlikely to be involved in the atherogenic process in arteries, and supports our findings that *H. pylori* does not influence the interaction effect of *TLR4* rs11536889 and *MyD88* rs7744 in CAD risk.

Our study has some limitations. Firstly, although our study comprised 424 CAD participants and 424 controls, this sample size may still be relatively insufficient for fully analyzing interaction effects. Secondly, additional adenosine functional tests were absent, so we could not investigate the relationship of SNP-SNP interaction effects on microvascular dysfunction in the participants [43]. Thirdly, some information was lost for a small number of study participants, such as lifestyle factors (*i.e.*, smoking and alcohol status), precluding their use as environmental factors in our multivariate logistic regression. Lastly, this study was hospital-based, which might increase the selection bias in comparison with population-based study.

## 5. Conclusions

In summary, our study is the first to show that a novel SNP interaction between *TLR4* rs11536889 and *MyD88* rs7744 is associated with an increased risk of CAD. Furthermore, the interaction strength was enhanced under conditions of hyperglycemia or hyperlipidemia. Our results provide a potential genetic clue to help predict CAD risk in susceptible people. Large-scale studies and experiments to determine the mechanisms are required to confirm the findings of this study.

## Figures and Tables

**Table 1:** Baseline characteristics of the study participants.

Variability	Cases	Controls	*p* Value
Total			
Age (year)	59.47 ± 10.81	59.27 ± 10.89	0.790
Sex			
Male (%)	269 (63.4)	258 (60.8)	0.436
Female (%)	155 (36.6)	166 (39.2)	
*H. pylori*			
Positive (%)	183 (43.2)	185 (43.7)	0.866
Negative (%)	241 (56.8)	239 (56.3)	
Smoking			
Yes (%)	166 (39.2)	124 (29.2)	0.090
No (%)	257 (60.6)	247 (58.3)	
Missing (%)	1 (0.2)	53 (12.5)	
Alcohol consumption			
Yes (%)	63 (14.9)	67 (15.8)	0.230
No (%)	361 (84.9)	304 (71.7)	
Missing (%)	1 (0.2)	53 (12.5)	
Hypertension			
Yes (%)	291 (68.6)	244 (57.5)	**0.001**
No (%)	133 (31.4)	180 (42.5)	
Hyperglycemia			
Yes (%)	165 (38.9)	101 (23.8)	**0.000**
No (%)	259 (61.1)	323 (76.2)	
Hyperlipidemia			
Yes (%)	227 (53.5)	186 (43.9)	**0.005**
No (%)	197 (46.5)	238 (56.1)	

Note: *H. pylori: Helicobacter pylori*.

**Table 2:** Two-way interactions between *TLR4* and *MyD88* polymorphisms in the risk of CAD.

*TLR4*	Genotypes	Number of Participants	*MyD88* rs7744			
			AA	AG + GG	AA + AG	GG
rs10116253	TC + CC	No. of controls/cases	123/101	179/169	258/242	44/28
		OR (95% CI)	1.0 (ref.)	1.052 (0.599–1.848)	1.0 (ref.)	0.715 (0.427–1.197)
	TT	No. of controls/cases	51/56	71/98	101/128	21/26
		OR (95% CI)	1.158 (0.608–2.207)	1.561 (0.844–2.887)	1.313 (0.951–1.812)	1.406 (0.762–2.592)
			*p* = 0.654, OR (95% CI) = 1.148 (0.627–2.104)		*p* = 0.322, OR (95% CI) = 1.517 (0.665–3.463)	
	CC	No. of controls/cases	31/29	51/42	67/67	15/4
		OR (95% CI)	1.0 (ref.)	0.859 (0.433–1.702)	1.0 (ref.)	0.338 (0.103–1.106)
	TC + TT	No. of controls/cases	143/128	143/225	288/303	54/50
		OR (95% CI)	0.982 (0.558–1.728)	1.290 (0.739–2.252)	1.078 (0.735–1.581)	1.121 (0.651–1.931)
			*p* = 0.347, OR (95% CI) = 1.423 (0.682–2.966)		*p* = 0.065, OR (95% CI) = 3.231 (0.929–11.236)	
rs10983755	GA + AA	No. of controls/cases	94/76	141/130	203/184	32/22
		OR (95% CI)	1.0 (ref.)	1.110 (0.749–1.644)	1.0 (ref.)	0.774 (0.429–1.395)
	GG	No. of controls/cases	80/81	109/137	156/186	33/32
		OR (95% CI)	1.214 (0.781–1.887)	1.595 (1.061–2.396)	1.292 (0.959–1.741)	1.139 (0.667–1.945)
			*p* = 0.612, OR (95% CI) = 1.158 (0.657–2.042)		*p* = 0.728, OR (95% CI) = 1.153 (0.517–2.570)	
	AA	No. of controls/cases	14/14	24/22	30/35	8/1
		OR (95% CI)	1.0 (ref.)	0.890 (0.312–2.541)	1.0 (ref.)	0.125 (0.013–1.217)
	GA + GG	No. of controls/cases	160/143	226/245	325/335	61/53
		OR (95% CI)	0.997 (0.453–2.195)	1.287 (0.587–2.822)	0.947 (0.562–1.596)	0.908 (0.471–1.752)
			*p* = 0.777, OR (95% CI) = 1.156 (0.424–3.157)		*p* = 0.074, OR (95% CI) = 7.346 (0.823–65.536)	
rs11536889	GG	No. of controls/cases	100/99	170/156	224/221	46/34
		OR (95% CI)	1.0 (ref.)	0.930 (0.653–1.323)	1.0 (ref.)	0.793 (0.485–1.297)
	GC + CC	No. of controls/cases	74/58	80/111	135/149	19/20
		OR (95% CI)	0.782 (0.501–1.220)	1.395 (0.934–2.085)	1.123 (0.828–1.522)	1.258 (0.640–2.471)
			***p* = 0.024, OR (95% CI) = 1.928 (1.089–3.413)**		*p* = 0.436, OR (95% CI) = 1.399 (0.601–3.258)	
	GG + GC	No. of controls/cases	165/152	238/247	345/352	58/47
		OR (95% CI)	1.0 (ref.)	1.143 (0.854–1.529)	1.0 (ref.)	0.851 (0.558–1.298)
	CC	No. of controls/cases	9/5	12/20	14/18	7/7
		OR (95% CI)	0.589 (0.192–1.810)	1.884 (0.876–4.052)	1.237 (0.598–2.561)	1.113 (0.379–3.267)
			*p* = 0.119, OR (95% CI) = 2.943 (0.756–11.454)		*p* = 0.909, OR (95% CI) = 1.082 (0.280–4.181)	

Notes: All tests were adjusted by age, sex, hypertension, hyperglycemia, hyperlipidemia and *H. pylori* infection. Statistically significant interaction was highlighted in bold (*p* value for interaction <0.05). CAD: coronary artery disease; *TLR4*: toll-like receptor 4; *MyD88*: myeloid differentiation factor 88; OR: odds ratio; CI: confidence interval; ref.: reference.

**Table 3:** The effect of confounding risk factors on the interaction between *TLR4* rs11536889 and *MyD88* rs7744 in the risk of CAD.

*TLR4*	*MyD88*	Controls (*n*)	Cases (*n*)	Cases *vs.* Controls		Controls (*n*)	Cases (*n*)	Cases *vs.* Controls	
rs11536889	rs7744			OR (95% CI)	*p*			OR (95% CI)	*p*
		*H. pylori* (−) ^a^				*H. pylori* (+) ^a^			
GG	AA	55	53	1 (ref.)		45	46	1 (ref.)	
GG	AG + GG	95	86	0.954 (0.582–1.564)	0.851	75	70	0.963 (0.558–1.662)	0.892
GC + CC	AA	45	35	0.829 (0.456–1.510)	0.541	29	23	0.784 (0.386–1.592)	0.501
GC + CC	AG + GG	44	67	1.683 (0.959–2.956)	0.070	36	44	1.180 (0.609–2.287)	0.624
				*p* = 0.065, OR (95% CI) = 2.078 (0.956–4.517)				*p* = 0.354, OR (95% CI) = 1.531 (0.621–3.773)	
		Hyperglycemia (−) ^b^				Hyperglycemia (+) ^b^			
GG	AA	76	60	1 (ref.)		24	39	1 (ref.)	
GG	AG + GG	128	97	0.969 (0.626–1.501)	0.890	42	59	0.948 (0.488–1.842)	0.875
GC + CC	AA	54	42	0.967 (0.566–1.650)	0.902	20	16	0.477 (0.200–1.137)	0.095
GC + CC	AG + GG	65	60	1.240 (0.748–2.055)	0.404	15	51	2.265 (1.024–5.011)	0.044
				*p* = 0.417, OR (95% CI) = 1.336 (0.664–2.686)				***p* = 0.004, OR (95% CI) = 4.905 (1.640–14.673)**	
		Hyperlipidemia (−) ^c^				Hyperlipidemia (+) ^c^			
GG	AA	60	46	1 (ref.)		40	53	1 (ref.)	
GG	AG + GG	91	67	0.989 (0.594–1.647)	0.968	79	89	0.930 (0.551–1.571)	0.787
GC + CC	AA	36	32	1.152 (0.621–2.136)	0.653	38	26	0.569 (0.293–1.107)	0.097
GC + CC	AG + GG	51	52	1.390 (0.782–2.471)	0.261	29	59	1.613 (0.859–3.029)	0.137
				*p* = 0.726, OR (95% CI) = 1.156 (0.513–2.604)				***p* = 0.006, OR (95% CI) = 3.269 (1.398–7.644)**	

^a^, these tests were adjusted by age, sex, hypertension, hyperglycemia and hyperlipidemia; ^b^, these tests were adjusted by age, sex, hypertension, hyperlipidemia and *H. pylori* infection; ^c^, these tests were adjusted by age, sex, hypertension, hyperglycemia and *H. pylori* infection. Statistically significant interactions were highlighted in bold (*p* value for interaction <0.05). CAD: coronary artery disease; *TLR4*: toll-like receptor 4; *MyD88*: myeloid differentiation factor 88; *H. pylori*: *Helicobacter pylori*; OR: odds ratio; CI: confidence interval.

**Table 4:** The effect of confounding risk factors on the interaction between *TLR4* rs10116253 and *MyD88* rs7744 in the risk of CAD.

*TLR4*	*MyD88*	Controls (*n*)	Cases (*n*)	Cases *vs.* Controls		Controls (*n*)	Cases (*n*)	Cases *vs.* Controls	
rs10116253	rs7744			OR (95% CI)	*p*			OR (95% CI)	*p*
		*H. pylori* (−) ^a^				*H. pylori* (+) ^a^			
TC + CC	AA	69	62	1 (ref.)		54	39	1 (ref.)	
TC + CC	AG + GG	103	92	0.962 (0.430–2.153)	0.925	76	77	1.076 (0.507–2.285)	0.848
TT	AA	31	26	0.903 (0.361–2.260)	0.828	20	30	1.594 (0.657–3.868)	0.303
TT	AG + GG	36	61	1.825 (0.772–4.312)	0.170	35	37	1.123 (0.493–2.562)	0.782
				*p* = 0.082, OR (95% CI) = 2.032 (0.914–4.518)				*p* = 0.132, OR (95% CI) = 0.502 (0.205–1.231)	
		Hyperglycemia (−) ^b^				Hyperglycemia (+) ^b^			
TC + CC	AA	95	67	1 (ref.)		28	34	1 (ref.)	
TC + CC	AG + GG	137	102	1.041 (0.518–2.092)	0.910	42	67	1.384 (0.511–3.752)	0.523
TT	AA	35	35	1.243 (0.556–2.781)	0.597	16	21	0.955 (0.3026–3.023)	0.937
TT	AG + GG	56	55	1.349 (0.633–2.875)	0.439	15	43	2.744 (0.903–8.345)	0.075
				*p* = 0.863, OR (95% CI) = 0.938 (0.450–1.953)				*p* = 0.348, OR (95% CI) = 1.683 (0.567–4.992)	
		Hyperlipidemia (−) ^c^				Hyperlipidemia (+) ^c^			
TC + CC	AA	68	52	1 (ref.)		55	49	1 (ref.)	
TC + CC	AG + GG	102	71	1.026 (0.451–2.333)	0.951	77	98	1.215 (0.542–2.720)	0.637
TT	AA	28	26	1.217 (0.480–3.087)	0.679	23	30	1.165 (0.427–3.178)	0.765
TT	AG + GG	40	48	1.908 (0.758–4.807)	0.170	31	50	1.513 (0.624–3.667)	0.359
				*p* = 0.354, OR (95% CI) = 1.497 (0.637–3.517)				*p* = 0.688, OR (95% CI) = 0.837 (0.351–1.996)	

^a^, these tests were adjusted by age, sex, hypertension, hyperglycemia and hyperlipidemia; ^b^, these tests were adjusted by age, sex, hypertension, hyperlipidemia and *H. pylori* infection; ^c^, these tests were adjusted by age, sex, hypertension, hyperglycemia and *H. pylori* infection; Statistically significant interactions were highlighted in bold (*p* value for interaction <0.05). CAD: coronary artery disease; *TLR4*: toll-like receptor 4; *MyD88*: myeloid differentiation factor 88; *H. pylori*: *Helicobacter pylori*; OR: odds ratio; CI: confidence interval.

**Table 5:** The effect of confounding risk factors on the interaction between *TLR4* rs10983755 and *MyD88* rs7744 in the risk of CAD.

*TLR4*	*MyD88*	Controls (*n*)	Cases (*n*)	Cases *vs.* Control		Controls (*n*)	Cases (*n*)	Cases *vs.* Controls	
rs10983755	rs7744			OR (95% CI)	*p*			OR (95% CI)	*p*
		*H. pylori* (−) ^a^				*H. pylori* (+) ^a^			
GA + AA	AA	51	46	1 (ref.)		43	30	1 (ref.)	
GA + AA	AG + GG	81	71	0.972 (0.583–1.619)	0.913	60	59	1.409 (0.782–2.539)	0.253
GG	AA	49	42	0.950 (0.536–1.686)	0.862	31	39	1.803 (0.929–3.500)	0.081
GG	AG + GG	58	82	1.567 (0.931–2.640)	0.091	51	55	1.546 (0.847–2.823)	0.156
				*p* = 0.160, OR (95% CI) = 1.697 (0.812–3.547)				*p* = 0.249, OR (95% CI) = 0.608 (0.261–1.416)	
		Hyperglycemia (−) ^b^				Hyperglycemia (+) ^b^			
GA + AA	AA	73	52	1 (ref.)		21	24	1 (ref.)	
GA + AA	AG + GG	106	77	1.044 (0.655–1.664)	0.857	35	53	1.362 (0.644–2.881)	0.419
GG	AA	57	50	1.206 (0.709–2.054)	0.489	23	31	1.087 (0.478–2.471)	0.842
GG	AG + GG	87	80	1.397 (0.865–2.256)	0.172	22	57	2.407 (1.097–5.284)	0.029
				*p* = 0.922, OR (95% CI) = 1.035 (0.523–2.046)				*p* = 0.509, OR (95% CI) = 1.421 (0.501–4.032)	
		Hyperlipidemia (−) ^c^				Hyperlipidemia (+) ^c^			
GA + AA	AA	49	40	1 (ref.)		45	36	1 (ref.)	
GA + AA	AG + GG	76	55	0.904 (0.519–1.575)	0.722	65	75	1.419 (0.804–2.504)	0.228
GG	AA	47	38	0.979 (0.532–1.802)	0.946	33	43	1.509 (0.780–2.920)	0.222
GG	AG + GG	66	64	1.247 (0.708–2.198)	0.445	43	73	2.117 (1.163–3.854)	0.014
				*p* = 0.441, OR (95% CI) = 1.366 (0.617–3.025)				*p* = 0.852, OR (95% CI) = 0.925 (0.407–2.101)	

^a^, these tests were adjusted by age, sex, hypertension, hyperglycemia and hyperlipidemia; ^b^, these tests were adjusted by age, sex, hypertension, hyperlipidemia and *H. pylori* infection; ^c^, these tests were adjusted by age, sex, hypertension, hyperglycemia and *H. pylori* infection; Statistically significant interactions were highlighted in bold (*p* value for interaction <0.05). CAD: coronary artery disease; *TLR4*: toll-like receptor 4; *MyD88*: myeloid differentiation factor 88; *H. pylori*: *Helicobacter pylori*; OR: odds ratio; CI: confidence interval.

## Supplementary Materials

The following are available online at www.mdpi.com/1660-4601/13/3/278/s1. Table S1. Primer sequences and reaction conditions. Table S2. The genotype frequencies and HWE in this study.

## Abbreviations

TLR4: toll-like receptor 4
MyD88: myeloid differentiation factor 88
CAD: coronary artery disease
SNP: single nucleotide polymorphism
*H. pylori*: *Helicobacter pylori*
3′-UTR: 3′-untranslated region
GWAS: genome-wide association study
LPS: lipopolysaccharide
FetA: fetuin-A
MmLDL: minimally modified low density lipoprotein
PCR-RFLP: polymerase chain restriction-restriction fragment length polymorphism
OR: odds ratio
CI: confidence interval
HWE: Hardy-Weinberg equilibrium
Chr: chromosome
