# Supplementary material for: SNP-SNP Interaction between TLR4 and MyD88 in Susceptibility to Coronary Artery Disease in the Chinese Han Population
Source: Int J Environ Res Public Health. 2016 Mar 4;13(3):278. doi: 10.3390/ijerph13030278 (PMC4808941; doi:10.3390/ijerph13030278)
Supplement: Supplementary file 1 [file ijerph-13-00278-s001.pdf]

Supplementary Materials: SNP-SNP Interaction between TLR4 and MyD88 in Susceptibility to Coronary Artery Disease in the Chinese Han Population

Dandan Sun, Liping Sun, Qian Xu, Yuehua Gong, Honghu Wang, Jun Yang and Yuan Yuan

Table S1. Primer sequences and reaction conditions.

SNPs	Primer Sequence	Annealing Temperature	Restriction Endonuclease	Digestive Temperature	Product Size
rs10116253	F: GGGTGTAAAAGC CAGGTAGAGGAGGT R: TGGAAAGTAGCAA GTGCAATGTAAGT	63 °C	BsmAI	37 °C	TT: 470 + 131 bp<br>TC: 601 + 470 + 131 bp
					CC: 233 bp
rs10983755	F: TGCCAGAACATCAA GAACACAGAAAAG R: TGGAAAGTAGCA AGTGCAATGTAAGT	60 °C	TaiI	65 °C	GG: 342 + 121 bp<br>GA: 463 + 342 + 121 bp
					AA: 463 bp
rs11536889	F: GCAGGAAGGA AGTGGGATGAC R: TGTTTCTGAGG AGGCTGGATG	63 °C	TaaI	65 °C	GG: 391 bp<br>GC: 391 + 292 + 99 bp
					CC: 292 + 99 bp
rs7744	F: AGGGAGCCTAA CCATGTCCCTGA R: AGGGTCTGT CCTGGGGCACA	61 °C	FspBI	37 °C	AA: 291 bp<br>AG: 291 + 172 + 119 bp
					GG: 172 + 119 bp

Table S2. The genotype frequencies and HWE in this study.

Genotype	Cases	Controls	*p* Value of HWE	Chr (Chr Position)
TLR4 rs10116253 (T > C)			0.333	9 (117702042)
TT (%)	154 (36.3)	122 (28.8)		
TC (%)	199 (46.9)	220 (51.9)		
CC (%)	71 (16.7)	82 (19.3)		
T allele (%)	507 (59.8)	464 (54.7)		
C allele (%)	341 (40.2)	384 (45.3)		
TLR4 rs10983755 (G > A)			0.186	9 (117702392)
GG (%)	218 (51.4)	189 (44.6)		
GA (%)	170 (40.1)	197 (46.5)		
AA (%)	36 (8.5)	38 (9.0)		
G allele (%)	606 (71.5)	575 (67.8)		
A allele (%)	242 (28.5)	273 (32.2)		
TLR4 rs11536889 (G > C)			0.383	9 (117715853)
GG (%)	255 (60.1)	270 (63.7)		
GC (%)	144 (34.0)	133 (31.4)		
CC (%)	25 (5.9)	21 (5.0)		
G allele (%)	654 (77.1)	673 (79.4)		
C allele (%)	194 (22.9)	175 (20.6)		

MyD88 rs7744 (A > G)			0.177	3 (38142530)
AA (%)	157 (37.0)	174 (41.0)		
AG (%)	213 (50.2)	185 (43.6)		
GG (%)	54 (12.7)	65 (15.3)		
A allele (%)	527 (62.1)	533 (62.9)		
G allele (%)	321 (37.9)	315 (37.1)		

HWE: Hardy-Weiberg equilibrium; *TLR4*: toll-like receptor 4; *MyD88*: myeloid differentiation factor 88; Chr: chromosome.

© 2016 by the authors; licensee MDPI, Basel, Switzerland. This article is an open access article distributed under the terms and conditions of the Creative Commons by Attribution (CC-BY) license (http://creativecommons.org/licenses/by/4.0/).
